# Brain orexin promotes obesity resistance

**DOI:** 10.1111/j.1749-6632.2012.06585.x

**Published:** 2012-07-17

**Authors:** Catherine Kotz, Joshua Nixon, Tammy Butterick, Claudio Perez-Leighton, Jennifer Teske, Charles Billington

**Affiliations:** 1 Department of Veterans Affairs, GRECC and Research ServiceMinneapolis, Minnesota; 2 Food Science and Nutrition, University of MinnesotaSaint Paul, Minnesota; 3 Department of Medicine, University of MinnesotaMinneapolis, Minnesota; 4 Department of Neuroscience, University of MinnesotaMinneapolis, Minnesota; 5 Department of Nutritional Sciences, University of ArizonaTucson, Arizona; 6 Research Service, Southern VA Healthcare SystemTucson, Arizona

**Keywords:** orexin, obesity, spontaneous physical activity, nonexercise activity thermogenesis, energy expenditure

## Abstract

Resistance to obesity is becoming an exception rather than the norm, and understanding mechanisms that lead some to remain lean in spite of an obesigenic environment is critical if we are to find new ways to reverse this trend. Levels of energy intake and physical activity both contribute to body weight management, but it is challenging for most to adopt major long-term changes in either factor. Physical activity outside of formal exercise, also referred to as activity of daily living, and in stricter form, spontaneous physical activity (SPA), may be an attractive modifiable variable for obesity prevention. In this review, we discuss individual variability in SPA and NEAT (nonexercise thermogenesis, or the energy expended by SPA) and its relationship to obesity resistance. The hypothalamic neuropeptide orexin (hypocretin) may play a key role in regulating SPA and NEAT. We discuss how elevated orexin signaling capacity, in the context of a brain network modulating SPA, may play a major role in defining individual variability in SPA and NEAT. Greater activation of this SPA network leads to a lower propensity for fat mass gain and therefore may be an attractive target for obesity prevention and therapy.

## Obesity and individual variability

Obesity is a condition defined by a chronic excess of body fat^1^ and is positively correlated with shorter life expectancy, metabolic syndrome, type 2 diabetes, and coronary heart disease.^2^ Obesity has become a public health issue, as its incidence in adults and children has increased in the last two decades across both developed and underdeveloped societies.^3^–^5^

Humans show large variation in their susceptibility to obesity, which is determined by both environmental and genetic factors.^6^–^9^ A major factor in determining this variability is physical activity, and specifically a component of overall energy expenditure known as nonexercise induced thermogenesis (NEAT).^10^–^12^ NEAT includes all forms of energy expenditure not associated with formal exercise, such as standing and fidgeting.^10^,^13^–^15^ The complementary concept of spontaneous physical activity (SPA) is used to describe “any type of physical activity that does not qualify as voluntary exercise.”^16^,^17^ Both SPA and NEAT have a heredability component.^17^,^18^

SPA and NEAT are not interchangeable but are complementary concepts: NEAT refers to energy expenditure while SPA describes the types of physical activity that result in NEAT. Given the association between SPA and NEAT, it is not surprising that variability in SPA also contributes to variability in sensitivity to obesity. For example, lean people spend larger amounts of time standing (approximately two hours daily) than do obese people.^12^ While the contribution of NEAT to weight gain resistance may seem small, it can be significant. For example, one study showed that after overfeeding humans with 1,000 kcal daily for eight weeks, fat mass gain was significantly and negatively correlated with the increase in SPA and NEAT. Importantly, there was no change in volitional exercise, and no relationship between the observed change in fat mass and basal metabolism or postprandial thermogenesis.^11^

The neural mechanisms that underlie human variability in SPA are distributed processes involving multiple brain regions, neurotransmitters and neuropeptides, including cholecystokinin, corticotrophin releasing hormone, neuromedin, neuropeptide Y (NPY), leptin, and orexin (also known as hypocretin).^19^ While all are important, in this review we focus on the biological role of central orexin peptides and their receptors with respect to their role in obesity and obesity resistance.

## Orexin peptides and their receptors

The orexins are two closely related peptides, orexin A (OXA, hypocretin 1) and orexin B (OXB, hypocretin 2) that are produced by cleavage from a single propeptide.^20^,^21^ In mammals, the majority of CNS orexin peptides are synthesized in neurons located in the lateral hypothalamus and perifornical area. The hypothalamic orexin neurons are glutamatergic neurons with tonic firing, low-threshold spike on recovery from hyperpolarization and little spike adaptation.^22^,^23^ Recently, the existence of orexin neuronal subpopulations has been proposed based on morphological and electrophysiological evidence.^24^

The orexin peptides act through two G protein-coupled receptors, orexin receptor type 1 (OX1R, hypocretin receptor 1) and orexin receptor type 2 (OX2R, hypocretin receptor 2).^20^,^21^ Both orexin receptor subtypes can bind to OXA and OXB, but with differential affinity: OX1R has a higher affinity for OXA, while OX2R has equal affinity for either orexin peptide.^20^,^25^ Activation of both receptor subtypes leads to an increase in neuronal firing and an increase in intracellular calcium.^26^–^30^ Preadministration of the OX1R antagonist SB334867 can block OXA-induced SPA and NEAT,^16^,^31^–^34^ suggesting an important role for OX1R in mediating SPA and NEAT; however, OX2R involvement has not been ruled out.

An important characteristic of the orexin neurons are their projections to multiple brain regions.^35^–^39^ Neuroanatomical studies have shown that the orexin neurons have collateral projections within the CNS,^40^–^42^ transsynaptically collateral CNS efferents,^43^ or collateral efferents to both CNS regions and brown adipose tissue.^44^ The distribution pattern of the orexin receptors reflects the widespread projections of orexin neurons, as both orexin receptor subtypes are expressed throughout the brain. The orexin receptors show distinctive, yet overlapping patterns of expression, with a good agreement between mRNA and protein data.^45^–^50^ However, studies addressing colocalization of the orexin receptor subtypes at a cellular level are lacking. The wide distribution of the orexin receptors and orexinergic fibers initially suggested the orexin system was involved in multiple physiological processes, and current research supports a role for orexin in the control of arousal and sleep, reward, stress, and energy homeostasis.^51^–^56^

The main contribution of the orexin peptides to energy metabolism is elegantly exemplified in a mouse model that exhibits postnatal loss of orexin neurons.^57^ In these mice, the orexin promoter drives expression of the neurodegenerative gene ataxin-3, leading to progressive loss of the orexin neurons during development. These mice exhibit hypophagia, lower levels of SPA, and develop spontaneous onset obesity when fed a regular diet.^57^,^58^ These results suggest that one primary function of the orexin peptides is to drive energy expenditure, although they can also modulate food intake. Additional support for this idea comes from another mouse model in which the β-actin cytomegalovirus promoter drives overexpression of the orexin peptides.^59^ Consistent with the role of orexin in promoting energy expenditure, these mice show resistance to high-fat diet–induced obesity.^60^

## Orexin-mediated signaling

As discussed above, the orexin peptides exert their effects by binding to two closely related G protein-coupled receptors. *In vitro* and *in vivo* models show that orexin signaling is of an excitatory nature at the cellular level. Increased intracellular Ca^2+^ influx has been accepted as the most immediate cellular response to orexin receptor activation in both overexpression and *in vivo* models.^25^ Signaling responses for orexin receptors and their specific G-α subunit activation are currently under intense investigation. The activation of either orexin receptor can be coupled to G_q_, G_i/o_, or G_s_ G-α subunit proteins, which can modulate ion channels and exchangers to induce neuronal depolarization.^28^,^61^–^70^ Thus far, increased cellular activity by either OX1R and OX2R can be mediated by modulation of nonselective cationic currents (NSCC), voltage-gated calcium channels, the Na^+^/Ca^2+^ exchanger, and inwardly rectifying potassium channels.^27^,^28^,^30^,^71^–^79^

The type of intracellular mechanism triggered by activation of the receptors appears to be cell dependent. For example, in nucleus accumbens and nucleus of the solitary tract, OX1R/OX2R mediated depolarization requires a simultaneous decrease in K^+^ conductance and increase in NSCC.^26^,^73^ In GABAergic neurons from the arcuate nucleus, it occurs through a decrease in K^+^ conductance and activation of the Na^+^/Ca^2+^ exchanger.^80^ Finally, there are differences in the temporal profile of intracellular Ca^2+^ increases after OX1R/OX2R activation between neurons from the dorsal raphe and laterodorsal tegmental areas.^29^,^81^

The specific mechanisms involved in orexin mediated second messenger cascades, and their physiological relevance to obesity, are relatively undefined. Homogeneous overexpression models with human OX1R have revealed alterations in adenylyl cyclase activity via G_i/o_, G_s_, and G_q_ subunits but differ in their potency.^25^,^61^ OXA can also activate extracellular signal-regulated kinases (ERK1/2) and p38 mitogen-activated phosphate kinase (MAPK) in recombinant and adrenal cell culture models.^82^ OXA activation of either OX1R/OX2R in cells overexpressing either receptor can elicit the activation of ERK1/2 and p38 MAPK via multiple G-α subunits.^70^ Food deprivation in Wistar rats has also revealed differences in G-α subunit activation in hypothalamic tissue homogenates in response to OXA.^83^ However, discrete hypothalamic OXA-induced G-α subunit signaling responses for either OX1R or OX2R have yet to be determined when coexpressed.

The relevance of OX1R/OX2R in obesity has been exemplified in the obesity resistant (OR) and obesity prone (OP) rats. OR rats have higher basal levels of both intrinsic SPA and OXA-induced SPA following injections into the rostral lateral hypothalamic area (rLH) than OP or Sprague Dawley rats.^34^ Increased OXA sensitivity in OR rats appears to be due to an increase in receptor abundance compared to OP rats. While a difference in receptor density may address OXA sensitivity in OR rats, receptor functionality may also help explain the influence of orexin on SPA. Some aspects of orexin receptor sensitivity, distribution, and intracellular signaling mechanisms important in mediating OXA effects on SPA are currently under investigation using the OP or OR rat and other polygenic models of obesity. One such possibility under investigation is that that rLH orexin receptors in OR rats couple to G_s_ rather than G_i/o_ proteins, while the opposite occurs in OP rats.

## Orexin in an animal model of obesity resistance

Levin *et al.* showed that when fed a high-fat diet a tertile of outbred Sprague Dawley rats gained no more weight than chow-fed controls.^84^ These diet-induced obese rats and their weight-gain resistant counterparts, referred to as diet resistant, were selectively bred by a commercial vendor for over ten years,^85^,^86^ resulting in the current OP/OR polygenic model of obesity. The OP and OR rats have divergent weight gain profiles despite inconsistently observed differences in energy intake.^34^,^85^,^86^ While early studies demonstrated that obese rats had a dampened feeding response to satiety-promoting agents such as leptin^87^ and insulin,^88^ it was clear that other neural modulators as well as differences in SPA likely contributed to the polygenic obesity observed in OR and OP rats. As the orexins modulate SPA,^31^,^32^,^89^ these findings underscored the potential significance of orexin as a neural modulator regulating body weight in this rodent model.

Like the outbred diet-resistant rats, the selectively bred OR rats exhibit lower body weight and fat mass gain on a low-fat diet and gain less weight when fed high-fat diet relative to their obesity-prone counterparts.^33^,^34^,^85^,^90^ These OR rats consume significantly fewer absolute calories, but they consume statistically *more* calories when calculated on a per gram body mass basis.^34^ Differences in SPA between OP and OR rats suggested by an early study^91^ were confirmed by tracking SPA levels in several groups of OP and OR rats at various ages using a chamber that tracks activity in the *x*, *y*, and *z* axes using infrared beams.^34^,^90^,^92^,^93^ OR rats display more ambulatory and vertical movement independent of age or the presence of food,^34^,^90^,^92^ and this finding has been consistent across different groups of OR and OP rats.^92^ Subsequent studies revealed that this greater SPA was associated with greater energy expenditure,^92^ a lower propensity to gain fat mass throughout development, maturation, and aging,^90^ and maintenance of higher SPA levels after high-fat diet feeding.^33^

OXA-induced SPA is associated with a dose-dependent increase in energy expenditure.^31^ Together with our previous studies showing OXA-induced hyperphagia following rLH infusion,^94^,^95^ we hypothesized that heightened responses to SPA-promoting agents and a dampened response to feeding-stimulatory agents such as OXA would perpetuate the lean phenotype in OR rats. To test this, OR and OP rats with chronically implanted guide cannulae targeting the rLH were given graded doses of OXA. In separate experiments, SPA and food intake were measured postinjection in young and adult rats. As expected, OR rats had greater OXA-induced SPA independent of age,^34^ but OR rats also had greater OXA-induced food intake per gram body mass than OP rats (also independent of age).^34^ This increase in caloric intake fits with the above-described enhanced 24 hour basal caloric intake in OR versus OP rats, and the idea that any behavioral effect of OXA in OR rats may be heightened due to higher OXA signaling capacity in OR rats (described in more detail below). OR rats maintain a lean phenotype over time, suggesting that the negative caloric benefit of OXA-induced SPA appears to outweigh the positive calories due to OXA-induced hyperphagia. A potential contributing mechanism for this is the observed longer duration of OXA action on SPA relative to that on food intake.^89^ Further supporting the idea that OR rats have higher endogenous SPA, we later showed that OR rats are also more sensitive to other SPA-promoting stimuli including caloric restriction^96^ and appear to be intrinsically protected from treatments that lower SPA, such as high-fat diet feeding. We and others have shown that in contrast to OP rats, which display lower SPA levels after high-fat diet consumption, OR rats maintain high basal SPA levels and have greater OXA-induced SPA after high fat diet feeding.^97^ Most importantly, we also showed that rLH-OXA increases energy expenditure,^93^ and others found that daily OXA treatment reduces body weight by increasing SPA.^98^ These findings support the hypothesis that elevated energy expenditure due to SPA-promoting agents such as OXA and defense from SPA-dampening treatments protects against excessive adiposity gain in OR rats.

**Figure 1:**
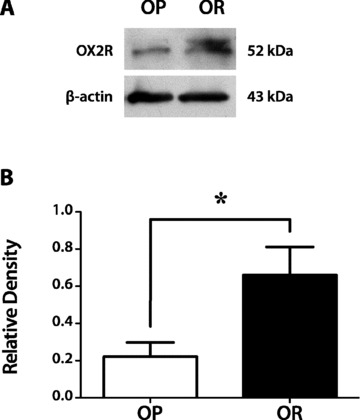
Difference in orexin 2 receptor (OX2R) protein levels between obesity-prone (OP) and obesity-resistant (OR) rats on chow diet. (A) Western blot analysis of caudal lateral hypothalamus (cLH) of OP and OR rats. The 52–53 kDA band is the predicted size of OXR2. Samples were normalized to β-actin. (B) Densitometric analysis of the Western blot data for all OP and OR rats. *N* = 4/group; *P < 0.05.

To understand whether differences in responsivity to OXA are driven by greater orexin signaling at the level of the peptide or the receptor, we analyzed mRNA data for prepro-orexin, OX1R, and OX2R from brain micropunches in OR and OP rats. Our data show that relative to OP rats, OR rats have greater orexin receptor mRNA in the rLH despite similar levels of preproorexin in the rLH or within whole hypothalamus.^34^ This elevated receptor mRNA is mirrored by elevated receptor peptide levels in OR rats (Fig. 1). We later showed greater orexin receptor mRNA in the dorsal raphe, locus coeruleus, and ventrolateral preoptic area, in addition to better sleep quality in OR rats, which would be expected to contribute to the favorable weight status observed.^99^ Together with our earlier work, these data suggest that elevated orexin receptor mRNA within distinct brain sites function to create a brain-wide orexin signaling network at the level of the receptor that perpetuates heightened basal and OXA-stimulated SPA levels, which attenuates adiposity gain in OR rats.

There are many animal models developed to amplify divergent locomotor activity, wheel running, or aerobic capacity.^17^,^100^ These models help clarify the role of overall physical activity level in obesity, but they do not mirror models varying in SPA, as exercise has been shown to have a strong motivational component, whereas SPA may not. A recent series of studies undertaken by Novak *et al.*^101^,^102^ in rodents selectively bred for high and low wheel running capacity (HCR and LCR, respectively)^103^ have shown that HCR rats also have greater SPA, and basal and activity-induced energy expenditure.^102^ Furthermore, like OR rats, HCR rats have heightened SPA following OXA infusion in the hypothalamic PVN.^101^ However, in contrast to OR rats, greater OXA-induced SPA in HCR rats is accompanied by greater OXA content but not increased orexin receptor mRNA in the perifornical lateral hypothalamus.^101^

It is possible that the differences in orexin function between animal models bred for high exercise and the OP and OR rats arise from long-term effects of high fat consumption in this particular model. In itself, this makes the OP and OR rats a more appropiate model for studies of diet-induced obesity. The evidence from the OP and OR model suggests that the interaction between high fat consumption and the orexins is determined by individual susceptibility to high fat consumption, which in turn might be determined by baseline orexin signaling through particular brain regions, including the rLH. In summary, while animal models of high exercise may share some of the same orexin signaling characteristics as that of high SPA models, there are likely differences in the regulatory control of orexin between these models, which might be a consequence of the high-fat intake used to derive the OP and OR rats.

## Orexin and sleep

As mentioned above, OR rats, with higher orexin signaling capacity, also exhibit more consolidated sleep relative to OP rats. The overall distribution of orexin fibers in the brain has suggested that the orexins play a role in a number of systems, including the maintenance of arousal.^104^,^105^ Orexin fibers have been shown to project to several brain nuclei implicated in the control of sleep state.^35^–^39^ Application of OXA in the locus coeruleus^104^,^106^ and lateral preoptic area^107^ of the rat increase wakefulness, primarily through a decrease in rapid eye movement (REM) sleep.^106^ Activity in locus coeruleus (LC) neurons increases following application of OXA.^104^–^106^ More recently, direct stimulation of electrical activity in orexin neurons using optogenetic techniques was shown to induce wakefulness in sleeping mice.^108^

In addition to projecting to sleep-wake nuclei, orexin cells receive input from brain systems involved in regulation of sleep-wakefulness. In mammals, circadian organization of activity including sleep-wake behavior is regulated by the endogenous clock located in the suprachiasmatic nucleus (SCN).^109^ Orexin cell bodies receive both limited direct contact from the SCN,^110^ as well as substantial indirect contact from the SCN via the medial preoptic area and the subparaventricular zone.^111^,^112^ Orexin neurons show circadian patterns of activation,^113^ and ablation of the SCN eliminates rhythmicity of orexin release.^114^ Introduction of chemicals known to increase arousal in rats, such as methamphetamines or the anti narcoleptic drug modafanil, increase nuclear Fos expression in orexin cell bodies.^115^,^116^ Furthermore, increasing the behavioral arousal of rats by sleep deprivation induced due to handling also increases the expression of nuclear Fos in OXA cells.^116^ Finally, in a diurnal rodent model, Fos expression patterns in orexin neurons are correlated with individual variation in the timing of daily wheel running activity.^117^ The orexins thus appear to be capable of both receiving information related to the arousal state of the animal, and relaying arousal information to other nuclei known to promote wakefulness.

The association between the orexins and arousal was strengthened by the discovery that sleep disorder narcolepsy is associated with a defect in the orexin system.^57^,^115^ While it is clear from this evidence that orexin is not necessary for wakefulness, data suggest that orexin is important in maintaining high levels of arousal, and that one major function of the orexin system is to stabilize sleep–wake transitions. Furthermore, there is a recognition that orexin activity may be incompatible with sleep, as direct activation of orexin neurons causes wakefulness in rodents,^108^ and silencing of orexin neuronal activity during the inactive period results in slow wave sleep.^118^

Two comorbidities associated with narcolepsy—cataplexy and obesity—help shed light on the importance of orexin in normal physiology. Early studies of orexin effects suggested that orexin results in the activation of motor activity,^119^,^120^ and it is well established that physical activity is correlated with both activation of orexin neurons 116,117 and increases in OXA release.^121^ In narcoleptic individuals, cataplexy (defined as a loss of muscle tone) is often triggered by emotional stress or physical exertion,^122^ and is preceded by a reduction of neuronal firing in the LC.^123^ Injection of OXA into the locus coeruleus activates LC neurons^104^–^106^ and increases muscle tone.^124^ Promotion of motor activity may thus be one important function of orexin, and this orexin-induced activity may be coupled strongly to behavioral state to maintain normal motor tone during periods of emotional or physical stress.

Obesity is a comorbidity of narcolepsy in both human and animal models.^57^,^122^,^125^ Both human and animal subjects with narcolepsy eat less as would be expected given the association between orexins and feeding behavior.^57^,^125^ Yet the effect of reduced caloric consumption is offset by decreases in physical activity, as narcoleptic individuals exhibit a significantly elevated body mass index relative to nonnarcoleptic patients.^122^ In human subjects, while the total time spent awake is not reduced, there is a decreased amplitude of circadian activity patterns, consistent with reduced overall physical activity.^126^ In a mouse model of narcolepsy, in which orexin neurons are ablated postnatally, physical activity during the active (but not the resting) phase is reduced in affected animals,^57^ and these animals subsequently become obese. These human and animal studies demonstrate that the effects of orexin on sleep–wake patterns and physical activity are consistent with the idea that orexin is a neuropeptide conveying resistance to obesity.

**Figure 2:**
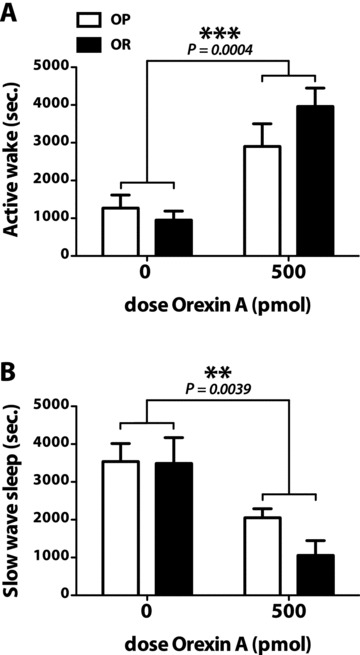
Total active wake (A) and slow wave sleep (B), in seconds, for obesity-prone (OP) and obesty-resistant (OR) rats for a 2-h period after treatment with vehicle or 500 pmol orexin A (OXA). In both panels, there is a significant overall effect of OXA on increasing active wake (*P* = 0.0004) and decreasing quiet sleep (*P* = 0.0039), respectively. However, there is no difference for either wake or sleep between OP and OR rats within treatment groups, despite the fact that OR rats show significantly higher levels of spontaneous physical activity following OXA treatment. *N* = 6/group; data are means ± SEM. **P < 0.005; ***P < 0.0005.

Instability of sleep patterns is known to contribute to weight gain.^127^,^128^ In this light, it could be argued that weight gain in narcolepsy is more due to disturbance of sleep than to reductions in activity due to lack of orexin signaling. However, evidence from a diurnal rodent model and from laboratory rats suggests that orexin-associated activity can be altered without disturbing total sleep. Wheel running activity in some Nile grass rats can occur exclusively at night, during the inactive phase, while in others wheel running follows the normal diurnal pattern.^129^ While Fos activation in orexin neurons occurs only during the light period in day-active animals, in night-active grass rats Fos is elevated both during nightly activity bouts and during the day.^117^ Behavioral measures of sleep in these animals showed that while the timing of sleep was changed in night-active animals due to wheel running at night, neither total sleep nor duration of sleep bouts differed between groups.^130^ Finally, previously unpublished data from our laboratory show that in OP and OR rats the effects of orexin on activity and arousal are not inextricably linked. Application of exogenous orexin significantly increases physical activity in OR relative to OP rats; however, recordings of total sleep using implanted EEG/EMG transmitters showed that this increase in activity was not caused by increased arousal or reduced sleep time in OR rats (Fig. 2). Importantly, while both sets of rats had increased time spent in wakefulness, there was not a greater increase in OR rats relative to OP rats. This lack of difference between OP and OR rats in time spent awake after orexin treatment suggests that SPA following orexin is not merely due to increased wakefulness; the SPA effect in OR rats is in addition to the wake-promoting effect of orexin.

## Translating SPA to energy expenditure

An important consideration is the relevance of SPA to overall energy expenditure. Energy expenditure (EE) comprises at least four main components, including basal metabolic rate (BMR; 60–70% of EE), which is the minimal energy required to maintain life, including heart beat, respiration, endocrine secretion, and kidney filtration. Diet-induced thermogenesis constitutes about 10% of total EE and includes energy related to the digestion, absorption, and metabolism of foods. Adaptive thermogenesis ranges from 10% to 15% of total EE and is related to adjustments in energy expenditure due to environment changes (e.g., shivering). Physical activity is the most variable of these components, ranging from 6% to 10% of total EE.^131^

SPA is neither a part of BMR nor a part of exercise physical activity. Therefore, it may be an attractive obesity target. SPA in humans was identified as early as 1954, and defined as a component of energy expenditure.^132^ Ravussin *et al.*^15^ showed that SPA in a human respiratory chamber averaged 348 kcal/day and, importantly, identified a large range in values: 100–700 kcal/day. Clearly, this wide range in values among humans suggests that there could be a large range in the weight gain response to overfeeding in humans, as demonstrated by Levine *et al.*^11^ Zurlo *et al.*^133^ showed that levels of SPA clustered in families, and could prospectively help explain propensity for weight gain in males, which suggests heritability of SPA. As discussed above, the idea that SPA level is an intrinsic heritable trait has recently been strengthened by Levine *et al.*, in his study showing that lean humans stand and ambulate for approximately two hours daily more than obese, which is not affected by weight loss or weight gain, in the obese and lean respectively.^11^ That spontaneous physical activity levels differ between mouse strains^134^ also suggests that SPA is an intrinsic, inherited trait that varies within animal species.

Despite large differences in body fat, energy intake, and body size, OR and OP rats expend a similar number of absolute kilocalories.^92^ This suggests that OR rats are less efficient in their calorie use, as they are expending relatively large amounts of calories to support their relatively smaller energy needs related to their reduced body circumference and body fat, which affect levels of heat loss. This supports the idea that elevated SPA and the resultant NEAT in OR rats contributes to their obesity resistant phenotype.^34^

When considering the therapeutic potential of SPA, a practical consideration is the comparability of the SPA difference between OP and OR rats to that in obesity-prone versus obesity-resistant humans. Calories expended via spontaneous physical activity correlate with whole body energy expenditure in humans and in animals.^14^,^92^ However, are the extra calories mediated by SPA in an OR rat, when compared to that in humans, enough to make meaningful changes in body weight? Based on indirect calorimetry studies, we estimate the energy flux in a rat to be about 100 kcal/day; SPA differences between OP and OR rats, when corrected for lean mass, amount to about 4 kcal/day or 4%.^92^ In humans, a 100 kcal energy gap (that level of energy intake above the daily requirement to maintain a stable body weight) per day, or 5% for a person in balance at 2,000 kcal/day, can lead to a 10-lb weight differential over one year.^135^ Based on this synthesis, we conclude that it is clear that SPA differences explaining obesity resistance in rodents can be relevant for human body weight control. Further, orexin-mediated mechanisms identified here may explain the differential body weight gain response to overfeeding in humans.^11^ For example, a prior human study showed that gain or loss of 10–20 pounds resulted in linear changes in energy expenditure, and that the majority of the change was specifically in nonresting energy expenditure (i.e., NEAT).^136^ We know that physical activity is correlated with both activation of orexin neurons^116^,^117^ and increases in OXA release,^121^ although the pathways through which this is effected are largely undefined. Thus increased SPA (either endogenous or artificial) could also lead to feedback mechanisms that maintain higher SPA levels in the future.

As discussed elsewhere in this review, orexin enhances feeding behavior and physical activity in a site-specific manner, with some sites conveying information regarding eating behavior, others activity, yet others both or neither. As our data and others show, the energetic consequence of these two behavioral outputs, when added up on a caloric basis, may result in negative energy balance and reduce body weight. In other words, the calories taken in by the effects of the orexin signal are outweighed by those expended via physical activity.

## Networks regulating spontaneous physical activity

**Figure 3:**
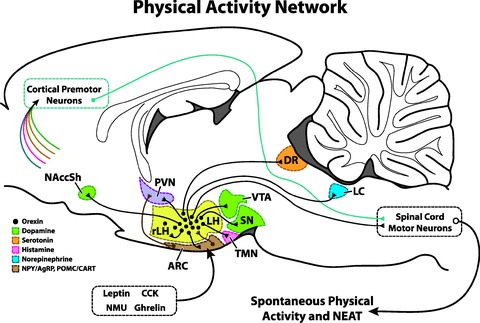
Overview of the involvement of orexin in a neural network regulating nonexercise activity thermogenesis (NEAT), indicating a sample of the brain areas, neuropeptides, and transmitters involved. Colors correspond to specific neuropeptide/hormone as follows: Black circles/projections: orexin; green, dopamine; orange, serotonin; pink, histamine; blue, norepinephrine; brown, neuropeptide Y/agouti-related protein (NPY/AgRP) and proopiomelanocortin/cocaine and amphetamine-related transcript (POMC/CART). Signals from all of these areas have the potential to influence cortical premotor neurons (indicated by arrows), and feedback from premotor neurons and orexinergic projections may interact to drive SPA. See the text for details. CCK, cholecystokinin; DR, dorsal raphe; LC, locus coeruleus; LH, lateral hypothalamus; NAccSH, shell of nucleus accumbens; NMU, neuromedin U; PVN, hypothalamic paraventricular nucleus; VTA, ventral tegmental area; rLH, rostral LH; SN, substantia nigra; TMN, tuberomammillary nucleus. Brain areas are not all to scale, and connections and neuropeptides/transmitters indicated are not all inclusive. Not all connections shown are discussed in this review. Figure modified from work by Kotz *et al*.^92^

Orexin plays a key role in an interdependent distributed brain network that regulates spontaneous physical activity. A significant number of brain sites that participate in this regulatory network in the forebrain and hindbrain have been identified, and there are a number of neurotransmitters that participate in this network, as depicted in Figure 3. The SPA network is distinct from the brain pathways that regulate purposeful activity, although the final common pathway leading to movement is clearly shared. Many of the brain sites that participate in the SPA network also participate in regulatory networks for food intake and other aspects of energy balance such as thermogenesis, but based both on distribution and on functional responses to stimulation, including orexin stimulation, it is clear that the SPA network is different from the networks that otherwise regulate energy balance.

Orexin is a unique contributor to this distributed SPA regulatory network by virtue of its position and projections. Orexin is made in one relatively small area of the hypothalamus, involving the caudal lateral hypothalamus and adjacent perifornical area.^20^,^21^ From these sites, orexin projects throughout the brain. Orexin neurons project throughout the hypothalamus, including the paraventricular, arcuate, rostrolateral, perifornical, and ventromedial areas, as well as to several extrahypothalamic sites, including the septal nuclei, bed nucleus of the stria terminalis, paraventricular, and reunions nuclei of the thalamus, zona incerta, subthalamic nucleus, central gray, substantia nigra, dorsal raphe nuclei, parabrachial nucleus, locus coeruleus, medullary reticular formation, area postrema, and nucleus of the solitary tract.^92^

The sites receiving orexin signal vary considerably with respect to the primary function associated with that site.^53^ Further, it is likely that in many sites the orexin effect on SPA is partially overlapping with other known orexin actions such as attention and wakefulness. While the functional outcomes of orexin action vary considerably from site to site, the production of SPA is common across many sites, and is widely distributed.^92^ This organization, involving a focused site of origin with wide distribution of effect, greatly enhances the potential potency of orexin as a regulator of spontaneous physical activity.

Orexin neurons receive input from a number of sites throughout the brain that are thought to influence expression of the orexin signal. The lateral hypothalamus receives afferents from cortical structures, including the prefrontal/orbitofrontal, insular, and olfactory cortices; limbic sites, including the amygdala, the hippocampal formation, and the shell of the nucleus accumbens; and from brainstem sites, including the nucleus of the solitary tract.^137^ Projections from other parts of hypothalamus include those from arcuate nucleus proopiomelanocortin (POMC)/cocaine and amphetamine-related transcript (CART) and NPY/agouti-related protein (AgRP) neurons.^138^,^139^ In addition, there is connectivity within the lateral hypothalamic area, notably projections from anterior to posterior portions.^140^ Whether all of these lateral hypothalamic projections play a significant role in regulating the activity of orexin neurons specifically is not yet determined, but a strong network of local lateral hypothalamic interneurons indicates the possibility of influences even by projections that do not directly synapse on orexin neurons themselves.^140^ Tracing studies have specifically identified projections to lateral hypothalamic orexin neurons from several regions of the amygdala, nucleus accumbens shell, bed nucleus of the stria terminalis, laterodorsal tegmental area, basal forebrain cholinergic neurons, GABAergic neurons in the preoptic area, and serotonergic neurons in the median/paramedian raphe nuclei.^138^

Orexin neurons are in a baseline intrinsic state of depolarized activity^141^ and are highly influenced by local conditions in an intralateral hypothalamic local network.^140^ The functional effects of the many afferents, and the associated neural function and neurotransmitters to orexin neurons, are underexploration. Application of the cholinergic agent carbachol activates many orexin neurons, indicating that the cholinergic input to orexin neurons from basal forebrain is excitatory.^138^ Cholinergic input from the laterodorsal tegmental area may also be excitatory to orexin neurons. Input of an as yet unidentified chemical type from the amygdala and bed nucleus of stria terminalis may also stimulate orexin neurons.^138^ CRF release from projections originating in the hypothalamic paraventricular nucleus also activates orexin neurons.^142^ Inhibitory influences on orexin neurons come from the preoptic area and from the serotonergic neurons in the median raphe.^138^

The physiological conditions that are known to affect orexin neurons include suppression of activity by glucose,^143^ along with prominent activation by hypoglycemia^144^ and by food restriction.^145^ There is evidence that low glucose states may be directly sensed by orexin neurons,^143^ although the likelihood of low glucose signals originating in other glucose sensing neurons with projections to orexin neurons is substantial. Intracellular Foxa2 signaling from the insulin receptor may be a mechanism allowing orexin neurons to sense the state of glucose and possibly short-term nutrition.^146^ Orexin neurons also receive local projections from leptin receptor bearing neurons, providing a means for translating the state of leptin signaling to the orexin neurons, and leptin action in lateral hypothalamus increases orexin action and decreases food intake.^147^ Leptin and energy state sensing arcuate neurons that express POMC/CART and NPY/AgRP also project to lateral hypothalamic neurons.^138^,^139^ A recent study has shown that amino acids, particularly nonessential amino acids, can stimulate orexin neurons directly through action on potassium channels and amino acid transporters.^148^ The stimulation provided by amino acids may be potent enough to overcome inhibition by glucose.^148^ Additional energy and nutrient related information may come to orexin neurons, perhaps directly, from the gut hormones ghrelin and glucagon-like peptide 1, both of which appear to activate orexin neurons in direct administration studies.^120^,^149^ Orexin neurons also receive input about physiological stress–related information, likely through a corticotrophin releasing hormone pathway.^142^ The integration of nutrition-related and other signals in orexin neurons, or in a network of which orexin is part, has not been defined.

Orexin signaling pathways can be further modified by the level of orexin-receptor expression in brain sites receiving orexin efferents. The conditions that lead to modulation of orexin receptor expression are incompletely defined. One example is the difference in orexin-receptor expression in a variety of brain sites associated with the difference between orexin-induced SPA response in polygenic OP and OR rat strains,^90^,^99^ as described elsewhere in this review.

Many brain areas contribute to SPA, and all of these areas operate in a network; thus activity in one area, affected by environmental cues or physiology as discussed above, influences firing patterns in other areas. Behavioral studies of SPA can determine the output of specific brain activity, improving understanding of the brain sites and neurotransmitter systems that are most important. The existing literature, however, is not always interpretable in a straightforward way. Locomotor activity measured in a beam-break chamber (as has been done for SPA measures) has also been used to assess nonspecific drug effects, as in studies of drugs of abuse. Similarly, low locomotor activity has been used as a diagnostic criterion for depression or illness in rats and mice.^150^ Thus, the data must be interpreted with care and in many cases repeated in a new context for full understanding.

Orexin A injected in almost all brain areas increases SPA, contrasting with feeding behavior that is stimulated after injection into only some of the same sites.^92^ The time course of action is different for the feeding and activity effects of orexin A, so the presence of one behavior (feeding or SPA) does not depend upon the other.^95^ Whether OXA-induced SPA is derived from orexin-enhanced wakefulness is not clear, but as discussed elsewhere in this review it appears likely that energy expenditure produced from SPA occurs after the initial waking event.

Many neurotransmitters have been shown to influence SPA in the network into which orexin action is projecting, although most of the available studies reporting on locomotor activity do not directly consider SPA itself, so at present some inferences must be made. Neurotransmitters that are likely to affect SPA include cholecystokinin, corticotrophin releasing hormone, neuromedin, NPY, leptin, and orexin.^19^ The current state of the evidence does not permit straightforward interpretation of the direction of effect for these neurotransmitters since there is disparate evidence based in part on site and type of administration.^19^ In general, it appears that in most situations each of these neurotransmitters can stimulate SPA, but orexin is the most consistent across all brain sites and types of stimulation. Little is presently known about the interactions of orexin with these other neurotransmitters with respect to the regulation of SPA.

Ultimately the output pathways for the SPA regulatory network must share engagement of the motor control pathways with voluntary movement brain mechanisms. The brain locations where these movement regulatory pathways begin to overlap have not yet been defined. It is likely that in part there are projections from forebrain structures, including the hypothalamus, to spinal motor neurons. An interesting possibility is that the SPA regulatory network engages cortical areas involved in voluntary motor control. The wide pattern of projections of the orexin neurons throughout the brain could mediate this function. In addition, a projection pathway from the accumbens through cortical premotor neurons and out to the spinal motor neurons has also been implicated.^92^

## Conclusion

Brain mechanisms mediate SPA and NEAT (Fig. 3), and the understanding of this concept is beginning to shed light on new ways to target obesity prevention and treatment. Studies of orexin and its role in obesity resistance show that stimulation of orexin receptors may be an attractive therapy for altering the course of excess body weight gain with aging, and also demonstrate that modulating SPA and NEAT has important consequences for obesity resistance. The knowledge of this can guide human obesity therapy immediately, as the option to include more low-level activity throughout one's day is likely more feasible advice to prevent and treat obesity than standard approaches that repeatedly fail over time. The greater challenge is in how to use information on brain SPA and NEAT networks to provide better pharmaceutical and/or other therapeutic approaches for treatment of obesity. The rapid development of new neurochemical methods of altering brain neurophysiology and recent advances in computer/brain interface technologies provide confidence that knowledge of brain SPA and NEAT networks could be therapeutically applied in the near future.
